# Process facilitators shifting between the support and expert roles in a complex work environment intervention in the Swedish healthcare sector

**DOI:** 10.1108/JHOM-10-2021-0382

**Published:** 2021-12-27

**Authors:** Ewa Wikström, Jonathan Severin, Ingibjorg H. Jonsdottir, Magnus Akerstrom

**Affiliations:** Department of Business Administration, School of Business, Economics and Law , University of Gothenburg , Gothenburg, Sweden; Region Västra Götaland , Institute of Stress Medicine , Gothenburg, Sweden; Institution of Neuroscience and Physiology , University of Gothenburg , Gothenburg, Sweden; Institute of Stress Medicine , Gothenburg, Sweden; Occupational and Environmental Medicine, Institute of Medicine , School of Public Health and Community Medicine , The Sahlgrenska Academy , University of Gothenburg , Gothenburg, Sweden

**Keywords:** Process evaluation, Work environment, Sickness absence, Process facilitators

## Abstract

**Purpose:**

Process facilitation as part of a complex intervention for changing or improving practices within workplaces is becoming a common work method. The aim of this study was to investigate what characterizes the process-facilitating role in a complex intervention.

**Design/methodology/approach:**

The present study focuses on a complex work environment intervention targeting eight organizational units (workplaces) in the Swedish healthcare sector. The study applies a mixed-method approach and has been carried out in two steps. First, a qualitative process evaluation was performed. Secondly, an evaluation was conducted to see to what extent these identified conditions and mechanisms affected the quantitative intervention effect in term of sickness absence.

**Findings:**

The analysis shows that the facilitating role consisted of three overlapping and partially iterative phases. These phases involved different activities for the facilitating role. Depending on how the facilitating role and the intervention were designed, various supporting conditions were found to significantly affect the outcome of the intervention measured as the total sickness absence.

**Research limitations/implications:**

It is concluded that the facilitation is not static or fixed during the change process. Instead, the facilitation role develops and emerges through the process of support during the different implementation phases.

**Practical implications:**

The facilitative role of performing support is based on a combination of support role activities and expert role activities. The support role focuses on support activities, while the expert role includes capacity building through knowledge- and legitimacy-oriented activities.

**Originality/value:**

This study contributes to earlier research by developing a methodological approach for carrying out process facilitation in complex interventions.

## Introduction

Organizational factors are of critical importance for work-related mental health ( Vingård, 2015 ). A health-promotive work environment benefits both employees and operations; it can increase job satisfaction and performance and reduce sickness absence ( Björk Brämberg *et al.* , 2018 ; Lohela-Karlsson *et al.* , 2015 ). To accomplish a health-promotive work environment, systematic work environment practices based on organizational-level measures have been recommended in the literature ( Burgess *et al.* , 2020 ; Cox *et al.* , 2007 ; Hellman *et al.* , 2019 , 2020 ). However, evaluations of organizational-level interventions have shown varying results ( Gray *et al.* , 2019 ; Montano *et al.* , 2014 ; Ruotsalainen *et al.* , 2014 ; Semmer, 2006 ), and information on how such interventions should be designed and carried out in practice is scarce ( Burgess *et al.* , 2020 ; Karanika-Murray *et al.* , 2016 ; Nielsen and Randall, 2013 ).

A key factor for successful improvement of the work environment is involving the overall strategic management in the organization. Another key factor is the involvement of human resources (HR) and the occupational health service (OHS) as supportive resources at a strategic level, and the collaboration between these two resources ( Schmidt *et al.* , 2017 ). In the traditional way of managing work environment initiatives, few models and processes for prevention and promotion at the organizational level are available. This has hindered work environment initiatives from an organizational perspective, highlighting the importance of collaboration between different managers and staff involved in managing the work environment ( Schmidt *et al.* , 2017 ; Liff *et al.* , 2017 ). Developing a more integrated way of working has the potential to provide support to the line managers and thus to facilitate the process of managing the work environment.

The function of line managers in the public sector has undergone major changes over the past 20 years, leading to increased responsibility and increased administration ( Forsell and Ivarsson Westerberg, 2014 ; Björk *et al.* , 2011 ). Despite clarification of responsibilities in the work environment and an increase in control systems, sickness absence due to work-related stress among employees in the public sector has not decreased. Changing formal descriptions of responsibilities and assignments or introducing more control and reporting systems does not seem to influence rates of sickness absence among employees in the public sector. Previous studies show recurring problems with developing systematic work environment management, especially regarding prevention and health promotion initiatives ( Liff *et al.* , 2017 ). Promotive or preventive work environment initiatives can, to a greater degree than the traditional, individual rehabilitative efforts, be assumed to require a process-oriented organizational perspective involving strategic management. One way of working with a process-oriented organizational perspective is to include a facilitator role and ensure that sufficient support structures are strategically involved.

As Langley and Denis (2011) showed, the different stakeholders' interactions and perspectives are of critical importance in managing an intervention. Dogherty *et al.* (2010) highlighted that previous researchers have treated the facilitator role as a “fixed” role during an intervention, and that there is a need for further research on the facilitator role from a processual perspective to provide a better understanding of the contexts and development of the role during the process. Harvey *et al.* (2002) and Dogherty *et al.* (2010) also suggested the need for further studies on what facilitators actually do to enable changes and how facilitation may be used. This is an important practical question because knowledge of general factors of collaboration and integration of work environment practices unfortunately does not reveal much about how and why any specific process support initiative does, or does not, enable changes, or how facilitation may be used in a specific context. Previous research [see, e.g. Grol and Grimshaw (2003) and Ferlie *et al* . (2005) ] has shown that formal evidence on best practices is not enough to ensure their implementation. Several studies note that more knowledge is needed on how facilitation as a process and a role works in different contexts to understand which facilitation mechanisms can prevail and enable change in a specific context ( Harvey *et al.* , 2002 ; Dogherty *et al.* , 2010 , 2012 ; Stetler *et al.* , 2006 ; Berta *et al.* , 2015 ; Lessard *et al.* , 2016 ).

The present study investigates the facilitator role and process within an intervention that was initiated in 2017 as a budget reinforcement in the county council of Region Västra Götaland in Sweden, with the aim of decreasing sickness absence and improving the work environment for the employees. As a part of this intervention, eight organizational units with high levels of sickness absence (>10%), in combination with a high employee turnover, were offered support through a facilitation process ( Akerstrom *et al.* , 2021 ). The purpose of the facilitation process was, in collaboration with management and individual managers, to identify underlying causes of work-related stress and contribute to improving these underlying causes by supporting ongoing work environment practices or new practices and thereby improve both employees' and the organizational units' health and well-being. The intervention was found to have an overall positive effect on both sickness absence and employee turnover. However, the results also showed a large variation in intervention effect between the participating intervention groups, which could not be explained by fidelity to the intention underlying the intervention ( Akerstrom *et al.* , 2021 ).

### Facilitation as a role and process

To understand how facilitation may be used in different contexts, Harvey *et al.* (2002) emphasized the importance of distinguishing between facilitation as a role and facilitation as a process. Facilitation as a role includes discrete task-focused activities, whereas facilitation as a process focuses on enabling individuals, teams and organizations to change. This is in line with Dogherty *et al.* (2010) , who highlighted that facilitation is often regarded as a central strategy to support and enable practitioners to translate evidence-based knowledge into practice and to improve practice. They concluded that facilitation could be defined as a combination of a role and a process involving individuals and groups, and that it is crucial to tailor the facilitation to the local context. Dogherty *et al.* (2012) highlighted facilitation as a multifaceted process and a team effort in which communication and relationship building are key components, whereas Stetler *et al.* (2006) defined it as a process of interactive problem-solving and support in interpersonal relationships. Berta *et al* . (2015) described facilitation as a guided interactional process frequently used in healthcare. They noted that its popularity is rooted in its potential to support uptake and application of scientific knowledge that stands to improve clinical and managerial decision-making, practice and, ultimately, patient outcomes and organizational performance. Moreover, they argued that the potential of facilitation could be reached if it stimulates meta-learning and relates the learning to organizational processes and work routines. They concluded that the facilitator role is to provide facilitation which acts as a learning mechanism. Lessard *et al.* (2016) emphasized that facilitation can be used as an approach to support practice change.

However, previous research on facilitation to support practitioners in improving practices and/or implementing new practices in healthcare has shown only modest effects, plausibly due to the complexity of the organizational context. An example of such research is Bååthe's study ( 2015 ), showing that obstacles in implementing new practices are related to both communication and collaboration. This is in line with a large number of studies that have examined observed and self-reported obstacles to new structures and work methods among different professional groups in healthcare organizations. In the same way, Langley and Denis (2011) have described contextual barriers, paying great attention to shortcomings in interpersonal relationships, role perceptions, communication patterns and teamwork. From the perspective of facilitation as a role, and as facilitating intervention processes, new practices and organizational changes, healthcare organizations have been defined as complex organizational environments where various professions work together, often under stressful conditions ( Langley and Denis, 2011 ).

To study the facilitator role and how the implementation of improvements and/or introduction of new organizational practices affects the work environment, theoretical frameworks are needed. The question of how to organize and facilitate an intervention and what to implement is not always so obvious ( May *et al.* , 2009 , 2016 ). How well complex interventions succeed in their implementation depends, among other things, on how well the intervention fits into the existing practices and social structures ( May *et al.* , 2016 ). May (2013) emphasized the importance of embedding the new practices through social mechanisms and group processes during the implementation.

Normative structures such as roles, rules and resources are integral parts of the workplace structure and will also affect how successful the implementation of the intervention is. In healthcare, professional hierarchies and subcultures are strong, and professional roles can be seen as socially constructed. As such, physicians and nurses are socialized into specific ways of thinking and acting that reflect the group's norms and basic assumptions. Therefore, highlighting collective measures can be beneficial because healthcare professionals depend on each other in their work, and hence are very dependent on each other in implementation processes.

Previous research on facilitation as a role and process has highlighted four central components: (1) meta-learning, (2) interactive problem-solving, (3) co-creation of intervention measures and (4) implementation of the developed practices and changes into practice through embedding the new practices into existing practices and social structures.

The present study focuses on the facilitator role and process within a complex work environment intervention in the healthcare sector and identifies the supporting and hindering factors in the facilitation process. The aims of the study were to investigate

1. what characterizes the facilitator role and process in a complex intervention,
2. the conditions under which, and mechanisms through which, the facilitator role and facilitation processes are effective in workplace interventions and
3. whether these conditions and mechanisms affect the intervention's effect on sickness absence.

The results from the study are intended to increase the knowledge of how preventive and promotive work environment processes can be developed through support using facilitation, and on the use of a facilitator role in a complex work environment intervention from a processual perspective.

## Study setting, materials and methods

### Setting and intervention design

The present study was carried out in the Swedish county council of Region Västra Götaland, which provides mainly health and dental care but also other services including culture, education, public transport and regional development. The county council has just over 55,000 employees.

**Figure 1:**
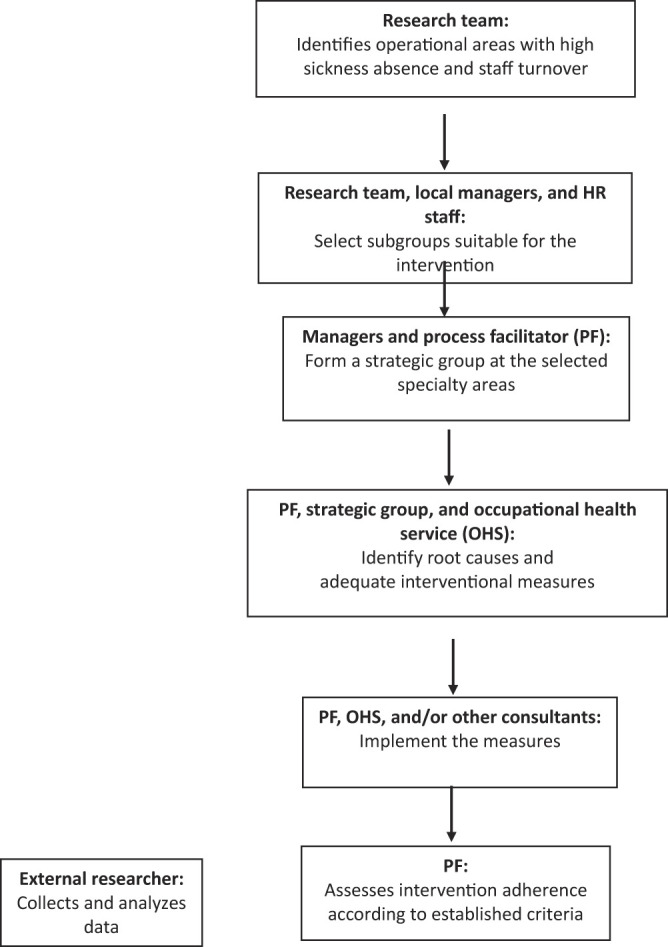
Overview of the intervention process ( Akerstrom *et al.* , 2021 )

The intervention ( Figure 1 ) has been described in detail elsewhere ( Akerstrom *et al.* , 2021 ). Briefly, it was initiated with an in-depth analysis of personnel and work environment statistics at all departments within the county council of Region Västra Götaland. The analysis identified eight operational areas (one of the organizational levels in the hierarchy, consisting of departments, operational areas and workplaces) with high sickness absence rates in combination with a high employee turnover. An external facilitator role (see below) was assigned to each identified operational area, and the management of these operational areas was consequently contacted and offered process support. Since the intervention could not include the entirety of the operational areas, separate organizational units (workplaces) within each operational area were selected to participate in the intervention ( Table 1 ). The participating units (i.e. intervention groups) were selected by the management of the operational area with knowledge of the units in question.

A strategic group consisting of managers and their HR partners at two or more hierarchical levels was formed. The investigation and analysis of the work environment and co-creation of intervention measures and work methods were performed by line managers and external support functions. The active involvement of the strategic group was also intended to ensure a good fit between the interventional measures and the local context.

Initial guiding principles for the co-creation of intervention measures were that the efforts were expected to affect the work environment, be deployed primarily at group and organizational level and include a clear and credible idea of mechanisms that could plausibly reduce sickness absence among the employees. Other guiding principles were that a committed strategic management group should be organized and that resources should be ensured for the process. Furthermore, the strategic management group should ensure that the efforts would be useful at the operative level and that they were linked to ongoing processes. The implemented measures ( Table 1 ) were chosen, depending on the context, by the strategic management, to create a consensus around the commitment and the process, and were implemented by the facilitator, the region's internal OHS, or external consultants.

The intervention ( Figure 1 ) in the form of support processes – using a method including both facilitation as a role and facilitation as a process – was based on the premise that managers and healthcare professionals are experts in their own environment. The researchers were not involved in the intervention and implementation process.

One starting point for the facilitation process was that changes in the work environment and new ways of working must, based on this expertise, stem from the aim that they (i.e. the changes) must be sustainable and meaningful. Changing work methods to improve the work environment also assumed that this could require a change in how managers and employees perceive their work environment. The facilitator role was managed by work environment experts with previous experience in facilitating processes (in total, four experts, responsible for supporting two processes each) from a research institute (the Institute of Stress Medicine (ISM)) and occupational health consultants, in most cases from the internal OHS. It was planned that, after about 2 years, the experts from the ISM would gradually withdraw their process support and facilitation and hand over this function to the managers, with or without the internal OHS as a process support function. The organizational units would thus, sometimes together with the internal OHS, take over responsibility for the continued process of improving and stabilizing the work environment.

### Analytical strategy

The analyses were carried out in two steps, using a mixed method approach. Firstly, a qualitative process evaluation was performed, using a thematic approach to investigate, from a processual perspective, what process support as a facilitating role means in a complex work environment intervention, and what conditions and mechanisms are decisive for whether the organizational units can benefit from the process support. Secondly, an evaluation was performed to determine to what extent these identified conditions and mechanisms affected the outcome of the intervention, measured as sickness absence. The analysis was performed using mixed effect models.

### Process evaluation

During the intervention, each facilitator was responsible for documenting the progress and important events or incidents (meetings, decisions, changes in key personnel, etc.) in a non-standardized log for each intervention group. In total, these logs, together with the facilitator's minutes of meetings, comprised about 30–40 pages per intervention group.

The process documentation was then compiled in a standardized format by one of the authors (J.S.). This was achieved by summarizing the content of the process documentation for each intervention group, using the following fixed categories: background, challenge, goal, course of events, initiation, context and sense of urgency, strategic group, measures (discussed, planned and implemented), critical incidents, key roles and the facilitator's perception of the process.

### Qualitative analysis

The qualitative data from the process documentation were analyzed using a thematic approach ( Miles *et al.* , 2014 ; Braun and Clarke, 2006 ). Initially, all documentation was read independently by all the authors to gain a sense of the whole. The documentation was read several times by the authors, and each author undertook tentative coding separately with a focus on the aim and keeping the research question in mind. For all the data sets, tentative codes were assigned according to content areas; then, meaning units were condensed and inductive codes were created. Initially, we labelled statements (notes and logs) in the documentation, and the descriptive content was categorized, with a focus on the patterns of statements and connections between them. Thereafter, the empirical categories were organized based on the theory of facilitation as a role and process. In this second coding step, we looked at the statement patterns and the connections between them to identify and interpret the importance of (1) meta-learning, (2) interactive problem-solving, (3) co-creation of intervention measures and (4) implementing the developed practices and changes in the workplace by embedding them into existing practices and social structures. This process resulted in an analysis of the documentation to identify themes and subthemes. These themes were discussed among all the authors and mirrored against the text and initial codes to ensure internal validity (i.e. credibility). The data were organized, according to the process view of the facilitators, into three main themes and their respective subthemes. The three main themes were named after the three phases of the intervention (“The pre-intervention phase,” “The intervention phase” and “The post-intervention phase”). The subthemes within the three main themes referred to the tasks and activities of the facilitators, as well as significant collaboration arenas or meetings during the process during the different phases. The main themes and subthemes demonstrate how the facilitator role evolved over time and underwent continuous change as an integral part of the operations of the organizational unit.

This method of interpreting the material allowed us to illustrate the use of facilitation in a complex work environment intervention from a processual perspective and to identify supporting and hindering conditions by quoting the process documentation. We thus achieved an overview of themes related to the process view of the facilitator role as the theoretical framework, including highlighting facilitation both as a role and as a process during the three main phases of the intervention. This way of conducting the analysis enabled us to highlight not only how the intervention was being implemented but also the facilitator role and the process during meetings, that is, how the role and process supported the implementation. The tasks and activities of the facilitator as well as significant collaboration arenas or meetings during the process and through the different phases generated the notes/logs that were used in the qualitative study. This created an understanding of how the facilitator role and process worked, as well as allowing for reexamination and further analysis of the subthemes ( Charmaz, 2006 ; Denzin and Lincoln, 2011 ).

### Impact of supporting and hindering conditions on the intervention effect

To assess how the supporting and hindering conditions, identified as outlined above, affected the quantitative outcome of the intervention in terms of sickness absence, monthly data from the region's administrative personnel system between January 2015 and October 2019 (i.e. covering the pre-, intervention and post-intervention phases) were obtained for each intervention group, together with aggregated data for the respective operational area and department (intervention groups excluded). Sickness absence was calculated as the absence percentages on a group level based on the number of hours of absence due to sickness, divided by the total number of hours the group was expected to work each month (vacation, parental leave and caring for sick children deducted). Furthermore, presence of the identified supporting conditions was assessed for each of the eight intervention groups separately using the process documentation. In cases where the presence of a supporting factor was not known or could not be assessed using the process information provided ( *n* = 4), supporting conditions were excluded for that specific intervention group. Lastly, the intervention groups were stratified for having all, some or none of the supporting conditions present.

### Statistical analysis

The impact of supporting and hindering conditions on the intervention effect was evaluated by adding a dummy variable for the presence of supporting conditions (e.g. all, some or none) as a fixed effect to a random intercept or random coefficient model (Proc Mixed in SAS, version 9.4; SAS Institute, Cary, NC, USA), with group and time (nested within groups) as random effects, as described by Akerstrom *et al* . (2021) . A first-order autoregressive correlation structure (AR(1)) was used to account for correlations between repeated measurements of the same group. Additionally, fixed effects for year (continuous) and month (categorical 1–12) were added to the model to control for time trends and seasonality, and an interaction term between the factor and a dummy variable for the intervention (0 up to the beginning of the intervention, thereafter 1) was added to analyze the effect. Furthermore, any concurrent effects for the reference groups (the respective operational areas and departments) were determined separately due to the structure of the collected data (i.e. data on a higher organizational level). Hypothesis testing for fixed effects was performed using Wald tests, and random effects were tested using likelihood ratio tests.

### Ethical approval

The study was approved by the Gothenburg Regional Ethics Review Board (Gothenburg, Sweden, reference number 911–18).

## Results

Descriptive information on the eight intervention groups is presented in Table 1 .

### What characterizes the facilitator role and process in a complex intervention?

**Figure 2:**
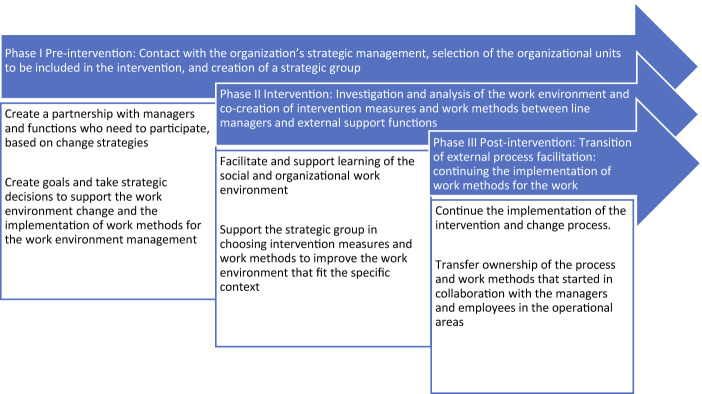
An overview of three overlapping and partially iterative phases (arrows) with the overall activities in each phase (boxes) in the design of the intervention

Our analysis shows that the facilitator role and process consisted of three overlapping and partially iterative phases (“The pre-intervention phase,” “The intervention phase” and “The post-intervention phase”). These three phases are illustrated in the schematic diagram in Figure 2 showing how the facilitator role and activities were designed for all organizational units that were part of the intervention. The phases are described in detail below.

We observed that the three different phases of the facilitation process involved different activities, content and social mechanisms for supporting the work environment intervention and change process. The facilitator role is characterized by activities that support the participants in embedding and enacting the new work methods.

Phase I, the initiation and pre-intervention phase, was aimed at the strategic management level and consisted selecting the units within the operational areas to be included in the intervention and creating a strategic group. A facilitating condition (i.e. a condition that promotes action aimed at developing the work environment and implementing the developed methods and changes into practice) for embedding and enacting the new strategies and strategic decisions in phase I was that the strategic management level perceived the issue of improving the work environment as important. Another important facilitating condition was that the strategic management group had sufficient knowledge of health-promotive aspects of the work environment and change processes. Hence, the strategic management felt that it was a priority to improve the work environment and prioritize prevention of work-related health problems. Therefore, the facilitator role in phase I involved putting emphasis on embedding the implementation through co-creation and commitment in order to support the enactment in the change process and find shared goals at the strategic management level. The facilitator role in this phase also involved activities to increase the strategic management team's understanding of the conditions and resources needed to create change in the work environment. In some of the workplaces, the facilitator role further included educational activities at the strategic level to support learning on work environment and change processes, such as enacting and embedding the new strategies and strategic decisions on improving the work environment in the overall strategic management plan. The educational activities were a part of the intervention. These activities involved knowledge building on the work environment, processual collaborative techniques and leadership in change processes, and on how the strategic management team could prepare for managing the facilitator role themselves. The educational activities are described in Table 1 . The educational activities were chosen based on what type of knowledge the different organizational units were missing in order to develop their work environment and implement the developed practices and changes into practice.

In phase II, the intervention phase, the facilitator role involved supporting the analyses of the work environment and supporting operational managers in preparing for and managing the change. It also involved support for the workplaces to make strategic choices regarding which intervention measures and work methods to choose to improve the work environment. The support also included support in conducting a collective analysis of the work environment related to the everyday operations at the unit and an investigation of how norms, roles and relationships in the workplaces can affect the work environment. In some of the workplaces, the facilitation role also included educational activities to support learning on processual collaborative techniques and models and how to manage change processes. This was intended to facilitate the enactment and to embed the new work methods within the social relations at the workplace.

The facilitator role in phase III, the post-intervention phase, which included a transition from external to internal process facilitation, involved focusing on change management and the transfer of ownership of the process and working methods that were started in partnership with the managers and employees at the operational level. As in phase II, in some of the workplaces it also included educational activities for operational managers to support their understanding and learning on how to facilitate the implementation of new work methods at the workplace. The educational activities aimed to support the operational managers in enacting and embedding the facilitator role in their role as operational managers.

The facilitator role and support process seem to have been effective in phase I, the outcome was different in phase II and few of the eight intervention groups succeeded in putting phase III into effect, where the workplaces, together with the OHS themselves, were supposed to take over responsibility for the change process and consolidate the new ways of working.

### What conditions and mechanisms are decisive for whether workplaces can benefit from the process support provided by the facilitator?

Within the present framework, the facilitator role was designed to address the conditions in a specific activity, which would enable flexible and adaptable work methods to be enacted and become embedded in the workplace ( Table 2 ). Consequently, depending on how facilitation and the intervention were designed together with the participating managers and employees, various facilitating and hindering conditions for enacting and embedding the new work methods were identified during the implementation process.

The intervention emerged from activities, and supporting conditions and mechanisms were identified in the three phases of the facilitation process. The eight intervention groups (workplaces) were consequently classified according to whether these conditions were present or not in the different phases. The following paragraphs describe these conditions during the three phases.

#### Phase I: pre-intervention phase

During the initiation of the intervention, four of the targeted intervention groups perceived the situation in their workplace as severe, due both to the fact that short- and long-term sickness absence had increased and to a sense of urgency among the managers at the strategic level concerning the need to change the work environment. Another supporting condition, found in four of the eight workplaces, was an existing good collaboration between HR and the OHS, which would be useful in implementing the intervention. Furthermore, five intervention groups were committed and had a positive attitude to receiving process support and using an intervention to improve the work environment, with good involvement and strategic management-level participation.

In organizations with good supporting conditions, it was easy to start up the processes in the operations. However, where supporting conditions were lacking, it was necessary to include educational activities to support the involved managers and staff to construct, enact and help embed the new strategies and strategic decisions. Hence, our analysis shows one condition in the support process that is central to the initial phase: supporting strategic managers in their leadership roles in preparing the implementation of the intervention measures. This supporting condition is facilitated by (1) good cooperation and trust between the OHS, HR and line managers, (2) good managerial involvement and strategic management-level participation and (3) a high sense of urgency.

Three out of the eight intervention groups were classified as having all the supporting conditions in phase I. Another three intervention groups were classified as having one out of the three supporting conditions and the remaining two intervention groups had none of the conditions ( Table 1 ).

#### Phase II: the intervention phase

During the intervention phase, a positive approach to receiving process support and implementing the improvement work from the operational manager was an important supporting condition in two of the intervention groups. This constituted a good basis for the change, which lasted throughout phases II and III, as reflected in the fact that there was a consensus and clear dialogue between everyone involved – the facilitator, the OHS as deliverer, HR, the strategic group, the managers and the employees. Therefore, one supporting condition that is central to phase II, which was identified was supporting operational managers in their leadership roles in initiating and managing the change. This change in the leadership role involved creating on-site commitment and inclusion of employees, good dialogue between managers and employees and engaged internal or external experts for co-creation and knowledge.

Besides the facilitating mechanisms of commitment, good dialogue and knowledge, it was also important that the organizational unit itself, before it was contacted by the facilitator, had performed a problem analysis. Having identified their problems helped the units understand that the work environment needed to change, and readied them to receive support (i.e. gave them a sense of urgency and commitment). Another important condition was that the workplaces had knowledge of the social and organizational work environment, as well as knowledge regarding process work and previous good experiences of driving change processes and implementation of new work methods. Where this knowledge was not present, the participants at the workplaces had to be supported in enacting and embedding the new work methods.

An analysis of the supporting conditions in phase II showed that two intervention groups had all the supporting factors in place, four intervention groups had some and the remaining two intervention groups had none of the supporting factors ( Table 1 ). Moreover, when we evaluated phase II in the eight intervention groups, we found that some groups showed good results, whereas restarts were needed in other groups, which slowed down the process. We concluded that one hindering condition that was central to whether the intervention groups would benefit from the facilitator role in phase II was lack of knowledge of the work environment and/or organizational change, or ambiguities in the organizational units about roles and responsibilities.

#### Phase III: the post-intervention phase – transition from external to internal process facilitation

To manage the change and obtain a sustainable result, it was vital to transfer ownership from the external facilitator to the organizational units themselves, with or without assistance of the OHS. As an example, one of the intervention groups chose to continue the initiated change process by continuing to focus on the desired goal, their vision for the future and the measures and changes that the manager and employees thought would be central to phase III: supporting operational managers and the OHS in taking over the facilitator role and continuing the change. This change of leadership was facilitated by a combination of all supporting conditions and facilitating mechanisms identified in phases I and II, namely, (1) good cooperation, (2) dialogue and trust, (3) knowledge of change management and the social and organizational work environment and (4) a high sense of urgency and commitment.

The analysis shows that only a few handovers of the facilitator role were conducted and only few real transitions took place, that is where the actual change process was handed over to the OHS and the workplaces themselves. Obstacles to a transition of the facilitator role are evident in several of the organizational units studied and included lack of knowledge of the work environment and organizational change, and ambiguities in many of the organizational units about roles and responsibilities, which hindered the termination of the external facilitator role.

This highlights the need to include educational activities on how to enact and embed the role of facilitation in the operational manager's role. The implications are, among other things, that the planning of measures to continue the change process takes time and delays the implementation of the work environment intervention. Other factors were low priority in scheduling of meetings, which causes the process to stop, and differences in views on the facilitator role between the organizational units and the OHS.

### Do supporting conditions and mechanisms affect the intervention's effect on sickness absence?

The presence of the identified supporting conditions (i.e. all, some or none of the supporting conditions present) in all three phases among the eight intervention groups was found to significantly affect the outcome of the intervention, measured as total sickness absence ( *p* = 0.001 for phase I, and *p* < 0.001 for all phases), showing that the intervention effect increased with the number of supporting conditions present ( Table 3 ). A corresponding change could not be seen among the corresponding reference groups for phase I ( *p* = 0.8 for operational areas, and *p* = 0.1 for departments), indicating that the observed effect among the intervention groups was attributed to the intervention in combination with the presence of supporting factors. For all phases, a significant effect was seen ( *p* = 0.02 for operational areas, and *p* = 0.03 for departments), but there was no significant association between the number of supporting conditions present and the size of the change in sickness absence.

## Discussion

In this study, we identify several supporting conditions that were important both for implementation and for how well the facilitation succeeded in supporting the process during different phases of a workplace intervention. These supporting factors were also shown to positively affect the outcome of the intervention, measured as total sickness absence. The results highlight the importance of ensuring that these supporting conditions enable not only complex work environment interventions and improvements, but also the daily systematic work environment management. Based on the results from the analysis of the processes and the facilitator role, it can be concluded that the working methods and facilitating mechanisms for a facilitator role, focusing on the support and expert roles during a complex work environment intervention and implementation of new practices, warrant discussion.

The results from the analysis moreover highlight that organizational units need to build up a capacity that supports the implementation of new work methods in the workplace. This capacity is important for benefiting from a complex work environment intervention. The process of building up this capacity needs to be supported by the facilitator. The capacity to enact and embed new work methods appears to require resources such as abilities; for instance, there must be an ability to work with the ideas and assumptions behind the change. It also seems important that the involved managers and staff recognize that the required change has the potential to add something new and that the change is relevant to the core operations in the daily work process.

These resources and abilities should, under ideal circumstances, already be in place as part of the systematic work environment management carried out by managers at the workplaces. Then, efforts to strengthen these resources could benefit all organizational units, regardless of the need to implement complex work environment interventions ( Dellve *et al.* , 2008 ).

This study contributes more detailed knowledge about the role of facilitation in achieving workplace improvements. In line with previous studies by Harvey *et al.* (2002) , Dogherty *et al.* (2010) , Stetler *et al.* (2006) , Berta *et al* . (2015) and Lessard *et al.* (2016) , the results from this study highlight that it is important to distinguish between facilitation as a role and facilitation as a process. However, the study also shows that the facilitator role needs to evolve during the different iterative phases of the intervention process. A methodological approach for process facilitators therefore needs to include both phase-specific activities and processes that enable change for individuals, teams and organizations. Examples of the intertwined relation between phase-specific activities and enabling/supporting change are:

1. Pre-intervention phase (phase I): support for the strategic managers initiating the implementation of the intervention and, if needed, expert advice to facilitate meta-learning through educational activities;
2. Intervention phase (phase II): support for operational managers in their leadership roles in preparing and managing the change, and, if needed, expert advice to facilitate meta-learning through educational activities; and
3. Post-intervention phase (phase III): support for operational managers and the OHS in assuming the facilitator role and continuing the change, and, if needed, expert advice to facilitate meta-learning through educational activities.

Accordingly, this study confirms the conclusions by Berta *et al* . (2015) , that including meta-learning which enables change is central to reaching the potential of facilitation in complex interventions.

However, previous studies report difficulties in reaching the potential of facilitation and sustaining the effects of interventions, and research on facilitation to support practitioners in implementing and improving new practices in healthcare has shown only modest effects ( Langley and Denis, 2011 ; Bååthe, 2015 ). Examples of obstacles highlighted elsewhere are shortcomings in interpersonal relationships, role perceptions, communication patterns and teamwork ( Bååthe, 2015 ). This study contributes to earlier research by describing phase-specific hindering conditions during the intervention process in six of the eight organizational units studied. Consequently, only two of the workplaces studied were found to have all the necessary supporting conditions and were therefore identified to have the potential to fully benefit from the facilitation process. This was also confirmed when analyzing the intervention's effects on total sickness absence.

Based on the results on hindering and supporting conditions and mechanisms, this study could contribute more knowledge in relation to the central questions identified by studies previously conducted by May (2013) and colleagues ( May *et al.* , 2009 , 2016 ), Langley and Denis (2011) and Wright and Zammuto (2013) . Examples of such questions posed in earlier studies are as follows: How should an intervention be organized and facilitated? How does the facilitator role emerge and develop? As previously mentioned, one of the central results of this study is the identification of phase-specific supporting conditions. In the pre-intervention phase, supporting conditions are characterized by central foci, a sense of urgency, as well as a trust and collaboration culture, and knowledge. During the intervention phase, the supporting conditions are characterized by commitment, dialogue and co-creation, and knowledge. In the post-intervention phase, the supporting conditions are characterized by clarity about responsibility and roles, a clear handover of the facilitator role, an understanding of working methods and knowledge. Moreover, altogether in our study population, the supporting conditions seemed to construct a capacity within the organizational units that enabled them to use the facilitator role to full potential. However, in workplaces that were identified as having several hindering conditions, the facilitator needed to shift between the facilitator role and the expert role to both facilitate the change process and, simultaneously through using an expert role, support the units to build the capacity for change. This study contributes to previous studies ( May *et al.* , 2009 , 2016 ; Langley and Denis, 2011 ; Wright and Zammuto, 2013 ) by elaborating a methodological process approach to the process facilitator role by phase-specifically balancing the facilitator role with the expert role.

Accordingly, good cooperation and trust between key managers and staff seem to construct a platform for the *capacity* to enact and embed new work methods in the organization during an implementation process, that is, a readiness to receive support. When this platform exists, the facilitator role can be defined as a support role for the strategic management level regarding preparation for the intervention and the implementation process. A supportive facilitator role seems crucial for operational managers because it is difficult to find new methods within an organization ( Severin *et al.* , 2021 ). Educational activities can add to this.

Organizations that have already built the capacity to manage implementation and change processes are usually characterized by good cooperation and trust between key managers in the process and staff. They also possess an ongoing method of analyzing potential work environment problems and perceive the work environment as an important issue to work with, and feel a sense of urgency regarding the need for change. Furthermore, this type of organization seems more committed and easier to support. It also seems that this type of organization has better knowledge of the work environment and positive experiences of driving change processes and implementing new work methods.

Conversely, we show that for organizations that have not built the capacity to manage implementation and change processes, different hindering conditions are evident during all the intervention phases. The absence of such capacity seems to create a fertile ground for different kinds of obstacles, such as lack of trust in collaborations between key managers and staff, in both vertical and horizontal structures. Also, this type of organization seems more frequently to have poorer knowledge of the work environment and of leadership and collaborative techniques during change processes. In this study, we have shown that the presence of these supporting factors is linked to the organizational outcomes of the intervention that is, intervention groups who had built this capacity gained a larger positive effect on the employees' sickness absence than did intervention groups lacking this capacity.

Depending on the organization's own capacity to manage implementation and change, the process support facilitator role will be defined differently from a processual perspective. In general, the facilitator role in an organization with the capacity to manage change processes could be performed by emphasizing the support role in the interaction with key managers and staff in the organization. Central to a support role is to support strategic managers in their leadership roles in preparing the implementation of the intervention, to support operational managers in their leadership roles in preparing the change and to support operational managers and the OHS in taking over the facilitator role and continuing the change.

**Figure 3:**
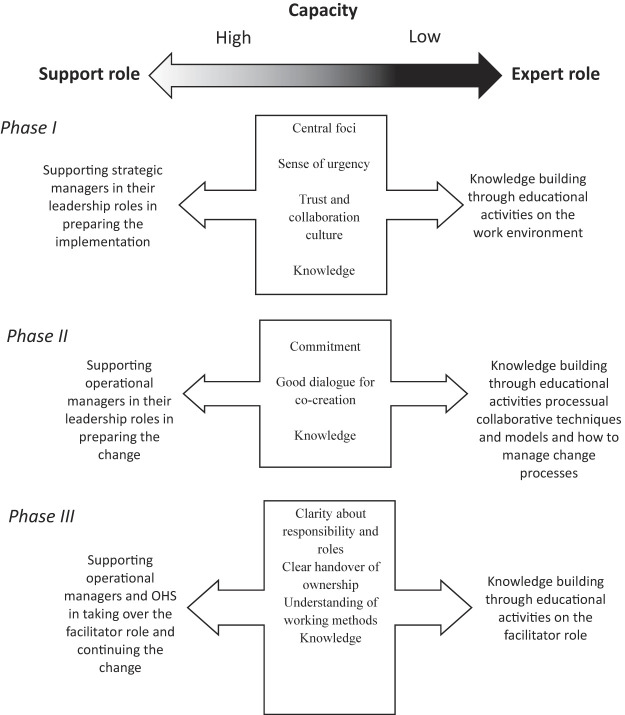
Interplay between the support role and the expert role in each intervention phase, depending on the capacity of the intervention group. OHS = occupational health service

By contrast, in organizations that have not built the capacity to manage implementation and change processes, the facilitator role needs to combine support role activities with expert role activities. An important type of expert role activity is knowledge building through educational activities on work environment, processual techniques and collaborative models, leadership in change processes and knowledge building through educational activities related to facilitation. In most organizations, the facilitator role needs to combine the support role with the expert role to succeed in performing process support during a complex intervention and change process. The expert role can then be described as supporting the organization's capacity building to increase capability, potential and the organization's contribution during the implementation and change process. The differences between these two types of roles are summarized in Figure 3 .

Another conclusion is that operational-level managers cannot build local capacity to enact and embed new work methods based solely on horizontal collaborations with support functions such as the OHS or HR. The managers need to have enough resources and decision-making latitude to succeed, and, therefore, strategic resources must also be included. In a top–down model, the intervention is initiated on a strategic level and then widens to include also the operational level. May's research on implementation and change work in healthcare has emphasized the importance of initially and, throughout a change process, building capacity to cope with the change work ( May *et al.* , 2009 , 2016 ).

Capacity building requires resources, which are linked to capability (i.e. the resources are linked to capability) – it must be possible to work based on the new structures and work methods, and it must be possible to include the process in everyday work. The organization must be equipped and able to work with the change processes, and all stakeholders must be able to see the potential in adding something new to the core operation. By evaluating the facilitator role from a processual perspective, information on the context and implementation of the intervention can be used to identify supportive and hindering factors that could explain the varying intervention effects that cannot be explained by fidelity ( Akerstrom *et al.* , 2021 ). These factors could consequently be used to optimize the design of future complex work environment interventions.

### Strengths and limitations

A strength of this study was the access to the process facilitators' own documentation about the planning and implementation context of the intervention in each intervention group. Another strength was the access to sickness absence data from the organizations' employee administrative system, in contrast to the self-reported data commonly used in effect evaluations. A limitation of this study was that the sickness absence data we had access to had been aggregated on a workplace level, which did not enable us to take into account changes caused by employee turnover. A further limitation of this study was that we could not use reference groups at the same organizational level as the intervention groups for the analysis of sickness absence. If such reference groups had been available, we could have made comparisons between intervention and reference groups within the same models instead of comparing the results of separate models. Although the conclusion of this study is transferable to other, similar complex interventions in healthcare organizations, further studies in similar contexts would be of interest. More thorough studies, including data collection through interviews and observations with managers and staff, would likewise be of interest and could provide a more detailed understanding of the extent to which the different supporting and hindering conditions are significant.

## Conclusion

The overall conclusion of this study is that the facilitator role in a complex work environment intervention is not static or fixed during the change process. Instead, the role develops and emerges through the process of performing support during the different phases of the intervention. The facilitator role of performing support is based on a combination of support role activities and expert role activities. The support role focuses on support activities, while the expert role includes capacity building through knowledge and educational activities to increase the organization's capability to work with the change process and to construct the potential to benefit from and contribute to the implementation process.

Our specific conclusions are threefold. Firstly, the intervention and change processes are dynamic processes involving different kinds of facilitator roles and activities being performed at the same time, and within the same process. Secondly, the facilitator role and process support build on an iterative relationship between the working methods and knowledge: the emerging working methods drive and constitute changes in work environment practices, while the new knowledge of the work environment and change management in collaborative processes stabilize and legitimize the changing work environment practice. Thirdly, our study also shows that intervention groups that have built this capacity achieve a larger positive effect on sickness absence compared to intervention groups lacking this capacity. Therefore, in a complex work environment intervention, the facilitator role and facilitative process need to shift between giving support and providing expert knowledge.

As for the practical implications, our study shows that the facilitator role not only has the potential to support changes in work environment practice, but, over time, this role may, by combining support and expert activities, also change the organizational unit's capacity to manage implementation of new practices and organizational change, and possibly also the organization's knowledge level on the work environment. This is particularly important in complex organizational settings such as healthcare organizations, that are characterized by a destabilized work environment and increased work-related illness.

## Figures and Tables

**Table 1:** Descriptive data on the intervention groups, measures performed and presence of supportive factors

	Intervention group							
	1	2	3	4	5	6	7	8
Type of workplace	Orthopedic surgery ward	Psychiatric hospital ward	Pediatric hospital clinic	Hospital service and maintenance unit	Pediatric hospital ward	Forensic psychiatric unit	Orthopedic aid and sterilization supply unit	Radiology hospital unit
Mean number of employees, *n* (range)^a^	99 (90–105)	41 (29–49)	191 (182–204)	289 (261–311)	164 (88–199)	268 (242–281)	131 (108–146)	458 (443–471)
Mean percentage of men in the organizational unit, %^a^	8	10	11	30	2	43	31	21
Measures performed during the intervention	Workshop on work culture	Workshop on improving work conditions	Occupational reflection groups	Coaching sessions for managers and management boards	Analysis of organizational conditions	Analysis of organizational conditions	Coaching sessions for managers	Coaching sessions for managers and the management board
	Support action plan implementation	Support action plan implementation	Coaching sessions for the management board	Analysis of organizational conditions	Training for mentors	Coaching sessions for the management board	Work environment training for employees	Workshop on the work environment for the management board
	Coaching sessions for managers	Coaching sessions for managers and the management board	Workshop on the work environment for a strategic group	Workshops and training	Work environment training for managers	Workshop on work culture	Workshop on roles and responsibility	Analysis of organizational conditions
	Ergonomic assessments				Training in work practices for employees			Training for employees
Presence of supporting factors^b^								
*Phase I*								
Good cooperation and trust between the OHS, HR and line managers	Yes	Yes	No	No	Yes	No	No	No
Good anchoring and strategic management-level participation	Yes	Yes	No	No	Yes	Yes	No	Yes
A high sense of urgency	Yes	Yes	Yes	No	Yes	No	No	No
*Phase II*								
On-site commitment including inclusion of employees	Yes	Yes	No	No	No	No	No	No
The organizational units themselves, before they were contacted by the facilitating role and process support, had conducted problem analyses	Yes	Yes	Yes	No	Yes	No	No	No
The organizational unit perceived that the work environment needed to change, and wanted support	Yes	Yes	Yes	No	Yes	Yes	–^c^	Yes
The organizational unit had knowledge of the social and organizational work environment	–^c^	Yes	–^c^	No	No	–^c^	No	No
The organizational unit had process knowledge and previous good experiences of driving change processes and of implementing new work methods	Yes	Yes	No	No	No	No	No	No
*Phase III*								
All supporting factors from phase I and phase II	–^d^	–^d^	–^d^	–^d^	–^d^	–^d^	–^d^	–^d^

**Note(s):** ^a^Mean number of employees during the study (58 months, January 2015–October 2019)

^b^Derived from the qualitative analyses of the process documentation

^c^Not known/not possible to assess from the process documentation

^d^See phase I and phase II

HR = human resources; OHS = occupational health service

**Source(s)** : The table is an extended version of the table from Akerstrom *et al* . (2021)

**Table 2:** The facilitator role and process were designed according to the conditions in the specific activity

The facilitating role and process	Phase I pre-intervention	Phase II intervention	Phase III post-intervention
The intervention was led by the facilitator role which was filled by external facilitators, all experts on organizational development and the work environment	Contact with the organization's strategic management, selection of the operational area that would be included in the intervention and creation of a strategic group	Investigation and analysis of the work environment and co-creation of intervention measures and work methods between line managers and external support functions	Transition of external process facilitation to continue the implementation of work methods for the work environment
To facilitate contextual adaptation and collective action, the intervention consisted of four components, all with theoretical underpinnings: Meta-learning, interactive problem solving, co-creation of the intervention measure and implementation of the developed practices and changes into practice	Phase I was directed toward managers in the strategic management group. Meetings were held to present and discuss the current work environment situation, investigate the interest in participating and gain more knowledge about the current state of the context. The aim was to create a sense of urgency and to start the process of co-creating the intervention and shared goal setting among managers in the strategic management group	In phase II, the operative line managers and staff were invited to participate	Phase III was the post-intervention phase during which, if the intervention had been successful, the implementation of the new work environment practices continued, and the practices were embedded in the workplace
Within this frame, the intervention was designed to be flexible and adaptive to allow for modifications. Consequently, the interaction between the facilitator role and process and the participants depended on how the latter responded to the goal of developing the work environment	Where needed, the facilitator role and process in phase I also involved educational activities to allow for dialogue to enable collective inquiry regarding perspectives and assumptions on the work environment, change processes and strategic management of change	Where needed, the facilitator role and process in phase II also involved educational activities to allow for dialogue to enable collective inquiry regarding perspectives and assumptions on the work environment, change processes and management of change related to participants' everyday practice and how their social norms, roles and relationships might influence the work environment	Where needed, the facilitator role and process in phase III also involved educational activities to allow for dialogue to enable collective inquiry regarding perspectives and assumptions on the work environment, change processes and management of change

**Table 3:** Estimated intervention effects on total sickness absence, by presence of the identified supporting factors

		Phase I					Phase I + II + III				
		*n* (groups)	Estimated effect^1^( *β* )	95% CI	*p* -value	Type 3 test *p* -value	*n* (groups)	Estimated effect^1^( *β* )	95% CI	*p* -value	Type 3 test *p* -value
*Presence of supporting conditions*											
	None	2	−0.35	(−2.1–1.4)	0.7	0.001	2	−0.24	(−1.9–1.5)	0.8	<0.001
	Some^2^	3	−1.7	(−3.2– −0.14)	0.03		4	−1.3	(−2.6–0.02)	0.05	
	All	3	−2.9	(−4.4– −1.4)	<0.001		2	−4.2	(−5.9– −2.4)	<0.001	

**Note(s):** ^1^Estimated difference (%) between pre- and post-intervention using mixed effect models

^2^One out of three for phase I, and two or three out of eight for phases I + II + III

CI = confidence interval
